# The fungal pathogen *Batrachochytrium salamandrivorans* is not detected in wild and captive amphibians from Mexico

**DOI:** 10.7717/peerj.14117

**Published:** 2022-10-03

**Authors:** M. Delia Basanta, Victor Avila-Akerberg, Allison Q. Byrne, Gabriela Castellanos-Morales, Tanya M. González Martínez, Yurixhi Maldonado-López, Erica Bree Rosenblum, Ireri Suazo-Ortuño, Gabriela Parra Olea, Eria A. Rebollar

**Affiliations:** 1 Centro de Ciencias Genómicas, Universidad Nacional Autónoma de México, Cuernavaca, Morelos, Mexico; 2 Facultad de Ciencias, Universidad Nacional Autónoma de México, Coyoacán, Ciudad de México, Mexico; 3 Department of Biology, University of Nevada Reno, Reno, Nevada, United States of America; 4 Instituto de Ciencias Agropecuarias y Rurales, Universidad Autónoma del Estado de México, Toluca, Estado de México, Mexico; 5 Department of Environmental Science, Policy, and Management, University of California, Berkeley, CA, United States of America; 6 Departamento de Conservación de la Biodiversidad, Colegio de la Frontera Sur Unidad, Villahermosa, Tabasco, México; 7 CONACYT-Instituto de Investigaciones sobre los Recursos Naturales, Universidad Michoacana de San Nicolás de Hidalgo, Morelia, Michoacán, Mexico; 8 Instituto de Investigaciones sobre los Recursos Naturales, Universidad Michoacana de San Nicolás de Hidalgo, Morelia, Michoacán, Mexico; 9 Instituto de Biología, Universidad Nacional Autónoma de México, Ciudad de México, Ciudad de México, Mexico

**Keywords:** Chytridiomycosis, Disease ecology, Amphibians, *B. salamandrivorans*

## Abstract

The recent emergence of the pathogen *Batrachochytrium salamandrivorans* (*Bsal*) is associated with rapid population declines of salamanders in Europe and its arrival to new areas could cause dramatic negative effects on other amphibian populations and species. Amphibian species, present in areas with high amphibian diversity such as Mexico, could be highly threatened due to the arrival of *Bsal*, particularly salamander species which are more vulnerable to chytridiomycosis caused by this pathogen. Thus, immediate surveillance is needed as a strategy to efficiently contend with this emerging infectious disease. In this study, we analyzed 490 wild and captive amphibians from 48 species across 76 sites in the North, Central, and South of Mexico to evaluate the presence of *Bsal*. Amphibians were sampled in sites with variable degrees of amphibian richness and suitability for *Bsal* according to previous studies. From the 76 sampling sites, 10 of them were located in areas with high amphibian richness and potential moderate to high *Bsal* habitat suitability. We did not detect *Bsal* in any of the samples, and no signs of the disease were observed in any individual at the time of sampling. Our results suggest that *Bsal* has not yet arrived at the sampled sites or could be at low prevalence within populations with low occurrence probability. This is the first study that evaluates the presence of *Bsal* in different regions and amphibian species in Mexico, which is the second most diverse country in salamander species in the world. We highlight the risk and the importance of continuing surveillance of *Bsal* in Mexico and discuss control strategies to avoid the introduction and spread of *Bsal* in the country.

## Introduction

Chytridiomycosis is an emerging disease caused by the fungal pathogens *Batrachochytrium dendrobatidis* (*Bd*) and *B. salamandrivorans* (*Bsal*) and is considered one of the principal causes of amphibian population declines worldwide (Longcore, Pessier & Nichols, 1999; Skerratt et al., 2007; Martel et al., 2013; Scheele et al., 2019; Fisher & Garner, 2020). Whereas *Bd* has been associated with worldwide population declines since the 1970s (Skerratt et al., 2007; Cheng et al., 2011; Fisher & Garner, 2020), the recent emergence of *Bsal* is associated with rapid population declines of the European salamander *Salamandra salamandra* (Martel et al., 2014; Stegen et al., 2017). Currently, the known distribution of *Bsal* is restricted to several countries in Asia and Europe (Martel et al., 2014; Laking et al., 2017; Yuan et al., 2018; Lötters et al., 2020). Amphibians from Asia have shown resistance and tolerance to *Bsal* infections, whereas European salamanders have suffered population declines and have shown severe symptoms due to *Bsal* infections (Martel et al., 2013; Martel et al., 2014). The tolerance or resistance to *Bsal* infections in Asiatic amphibians may be due to a long co-evolution between the pathogen and their hosts, whereas a recent introduction of this pathogen in naïve salamander populations could be the reason for the declines detected in Europe (Martel et al., 2014; Laking et al., 2017).

The pet trade has been suggested as one of the primary causes for the spread of *Bsal* to naïve areas, and experimental studies have demonstrated that *Bsal* infections are lethal to several North American salamanders such as *Notophthalmus viridescens*, *Chiropterotriton* spp. and *Aquiloeurycea cephalica* (Martel et al., 2014; North American *Bsal* Task Force, 2022). The pathogen *Bsal* has been detected in imported amphibians from Asia to Europe that did not show signs of disease (Cunningham et al., 2015; Gray et al., 2015; Nguyen et al., 2017; Yuan et al., 2018). Anurans have been considered more tolerant to *Bsal* infections than salamanders, acting as reservoirs and carrier species of the pathogen (Martel et al., 2014; Stegen et al., 2017; North American *Bsal* Task Force, 2022). However, recent evidence has shown that anurans can also be susceptible to *Bsal* infections under an experimental setting (Towe et al., 2021). Additionally, experimental co-infections of *Bd* and *Bsal* have shown higher mortalities than infections caused by only one of the pathogens (Longo, Fleischer & Lips, 2019). Considering the potential threat that *Bsal* represents for amphibian species in naïve regions, there is a considerable concern that *Bsal* will arrive in new areas such as North America, causing devastating impacts on amphibian diversity (Grant et al., 2015; Gray et al., 2015; North American *Bsal* Task Force, 2022).

Mexico ranks second in the world in the number of salamander species per country, and seventh in the number of amphibian species in general (AmphibiaWeb, 2022). Moreover, according to the International Union for Conservation of Nature (IUCN) Red List of Threatened Species, 62% of amphibian species in this country are in some category of risk (IUCN, 2022). Additionally, experimental studies of *Bsal* infections showed lethal effects (high susceptibility) in Mexican plethodontid salamanders (North American *Bsal* Task Force, 2022), thus more than 133 species of this group distributed in Mexico (AmphibiaWeb, 2022), could likely be susceptible to *Bsal* infection. It is highly likely that the potential introduction of *Bsal* to Mexico would represent a serious threat to local and global amphibian diversity. Previous studies have predicted that regions of Mexico such as the Trans Mexican Volcanic Belt, Sierra Madre del Sur, Sierra Madre Oriental, and Northern Oaxaca are places that could become highly suitable for *Bsal* (Basanta, Rebollar & Parra-Olea, 2019; García-Rodríguez et al., 2022). These studies also have found that the potential suitable habitat areas for *Bsal* corresponded to areas of high salamander diversity, increasing the risk of *Bsal*-vulnerable species losses in case the pathogen arrives (Basanta, Rebollar & Parra-Olea, 2019; García-Rodríguez et al., 2022). Thus, *Bsal* surveillance directed to areas of high suitability for *Bsal* and high amphibian diversity could be a good strategy to take immediate conservation actions. Recent efforts to monitor the presence of *Bsal* in the USA and Northern Mexico have not detected the pathogen (Bales et al., 2015; Klocke et al., 2017; Parrott et al., 2017; Newman et al., 2019; Hardman et al., 2020; Waddle et al., 2020; Hill et al., 2021). However, surveillance of *Bsal* presence in amphibians across Mexico is still lacking.

In this study, our main goal was to survey wild and captive amphibian populations in Mexico to search for the presence of *Bsal*. We sampled amphibians from different sites across the country with variable degrees of amphibian diversity and *Bsal* habitat suitability areas based on previous studies (Basanta, Rebollar & Parra-Olea, 2019; García-Rodríguez et al., 2022). This information could be informative for future surveillance efforts in areas of potential risk and identify new areas where pathogen arrival may be more likely to occur in Mexico.

## Materials & Methods

### Sampling design

We sampled wild amphibian species from North, Central and South Mexico. Additionally, we sampled individuals of *Ambystoma mexicanum* from a captivity center in Mexico City that includes different populations derived from pet owners and captive reproduction programs. All surveys took place between the years of 2015 to 2021. All wild and captive amphibians were captured using a new inverted plastic bag or sterile plastic container and manipulated them with a new pair of nitrile gloves to avoid cross-contamination (Phillott et al., 2010). Swab sampling was performed using standardized methods (Hyatt et al., 2007) and sterile swabs (MW113 rayon swabs, Medical Wire and Equipment, Corsham, UK). Collection permits were provided by the Secretaría del Medio Ambiente y Recursos Naturales (SEMARNAT) SGPA/DGVS/00947/16, SGPA/DGVS/03038/17, SGPA/DGVS/003513/18, SGPA/DGVS/002176/18, SPA-ENS/305/18, SEDUMA/SP/2738/2018, RBMM. DIREC/208/18, UNAM JJBIB/54/2017, SGPA/DGVS/5673/19, and SGPA/DGVS/02770/21.

### Molecular methods

Chytrid DNA was extracted from swabs using two extraction methods that are widely used for chytrid detection: Prepman Ultra and the Qiagen DNeasy Blood and Tissue Kit. For the Prepman extractions, we followed the protocol of Boyle et al. (2004) for 43 samples. Extractions from Prepman were diluted to a concentration of 1:10 to avoid any inhibition during the qPCR process (Boyle et al., 2004). Given that Qiagen extractions result in a higher quantity and quality of extracted DNA (Cheng et al., 2011), we switched for this approach for 447 samples and followed the protocol of the manufacturer with an additional lysozyme step (Rebollar et al., 2016).

We used the extracted DNA from swabs to detect *Bsal* through two different methods: quantitative TaqMan polymerase chain reaction (qPCR) as described in Martel et al. (2013), and an amplicon sequencing approach using the Fluidigm Juno system as described in Byrne et al. (2017) (Table S1). From 490 samples analyzed, 384 were tested with qPCR and 106 were sequenced *via* Fluidigm. For the qPCR, each sample was assayed in duplicate with one negative control (sterile water), one positive control (DNA extraction of *Bsal* isolate donated by Dr. Vance T. Vredenburg, San Francisco State University), and four standards of DNA made with a synthetic fragment of the 5.8S-ITS1 region of *Bsal* (Martel et al., 2013): 1,100,1000, and 10,000 ITS *Bsal* equivalent copies. We considered a positive detection of *Bsal* DNA if a detectable signal existed at 40 or fewer qPCR cycles. For the amplicon sequencing approach, samples were sequenced as described in Basanta et al. (2021a). Briefly, DNA extracts were first cleaned using an isopropanol precipitation and preamplified using two pools of 96 primer pairs. Samples were then loaded into a Fluidigm Juno LP 192.24 IFC (Fluidigm, Inc., South San Francisco, CA, USA) which performed microfluidic PCR amplification of 192 amplicons, one of which was designed to amplify the ITS1 region of *Bsal*. After amplification, samples were barcoded and pooled for sequencing on an Illumina MiSeq Lane using the Micro 300 bp paired-end kit.

### Salamander richness and *Bsal* suitability models

We estimated the amphibian richness for each site sampled. For this, we performed a richness map at a resolution of 30 arcseconds (∼1 km2) using the distribution range maps of amphibians from Mexico (IUCN, 2022) and the *fasterize* package (Ross, Sumner & Alliance, 2020) in R v.3.6.1 (R Core Team, 2019). Then, we extracted the richness values for each site using QGIS v3.8.3 (http://www.qgis.org). Amphibian richness can be used as a predictor of the diversity in the sampled sites and could inform how many species could be affected by *Bsal*. We also revised the risk category according to the IUCN (IUCN, 2022) for all amphibian species analyzed. Additionally, we estimated the potential risk of *Bsal* on each sampled site by extracting the *Bsal* suitability values from the model obtained in García-Rodríguez et al. (2022) using QGIS v3.8.3 (http://www.qgis.org). Because the logistic output of the suitability map of *Bsal* ranges from 0 to 1, with 0 indicating unsuitable habitat and 1 indicating the highest suitability, we also categorized them into four categories of suitability (not suitable, low suitability, moderate suitability, high suitability) according to Basanta, Rebollar & Parra-Olea (2019).

## Results

**Figure 1:**
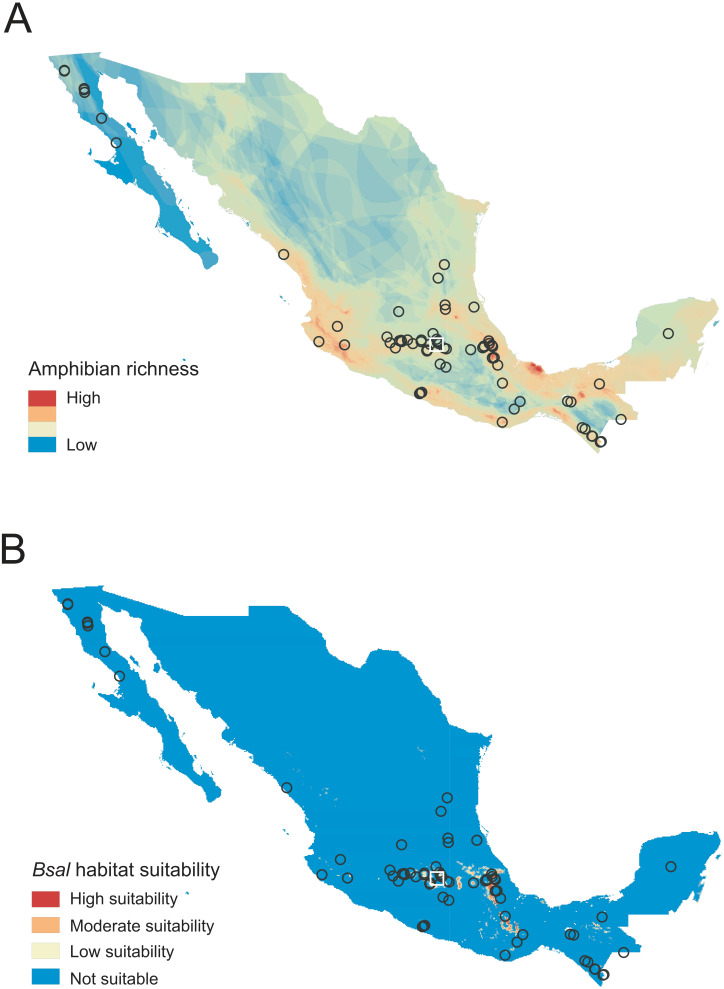
Sampled localities in Mexico analyzed for *Batrachochytrium salamandrivorans* (*Bsal*) detection. (A) Map of amphibian richness in Mexico constructed using amphibian distribution maps of the IUCN (2022). (B) Map of *Bsal* habitat suitability obtained from García-Rodríguez et al. (2022) and categorized following Basanta, Rebollar & Parra-Olea (2019). Circles represent sampled sites for wild amphibians, and the white square represents the sample site for captive amphibians.

We collected 490 samples of 48 species from nine families across 76 sites in Mexico (Fig. 1, Table 1, Table S1). Of the total number of samples, 463 were from wild individuals and 27 from captive individual, none were *Bsal*-positive by our laboratory methods (Table S1). No signs of the disease were observed in any individual at the time of sampling.

Of the 48 species sampled, 24 of them were endemic to Mexico, and according to the risk categories by the IUCN (IUCN, 2022), 23 are Critically Endangered (CR), Endangered (EN), Vulnerable (VU), or Near Threatened (NT) (Table S1). Our sampled sites in the wild were located in areas where 163 amphibian species are distributed (39% of the total amphibian diversity in Mexico). Of the 76 sampled sites in the wild, 50 were from areas of high species richness, hosting between 13 and 28 amphibian species (Fig. 1, Table S1). The rest of the sites (*n* = 26) were from areas of low-medium richness hosting between seven to 12 amphibian species (Table S1). Additionally, six species (84 of 490 [17%] individuals examined) were sampled from 10 sites with moderate to high *Bsal* habitat suitability. Finally, four sites had high species richness and moderate to high *Bsal* habitat suitability. The species surveyed in these four sites were *Ambystoma granulosum* (EN), *A. rivulare* (DD), *Chiropterotriton totonacus* (CR) and *Rana montezumae* (LC), which are distributed in the Trans-Mexican Volcanic Belt region and are all endemic to Mexico (Table S1).

**Table 1:** Sample size and taxonomic identification of amphibian species analyzed in this study to detect *Batrachochytrium salamandrivorans* in Mexico.

**Species**	***N***	**Order**	**Family**	**Predicted *Bsal* vulnerability**	***Bd* infection known in Mexico**	**References of *Bd* detection in Mexico**
*Anaxyrus boreas*	3	Anura	Bufonidae	Tolerant	Positive	Peralta-García et al. (2018) and Basanta et al. (2021a)
*Incilius sp.*	1	Anura	Bufonidae	Tolerant	Positive	Basanta et al. (2021a)
*Craugastor matudai*	2	Anura	Craugastoridae	No data	Positive	Basanta et al. (2021a)
*Craugastor pygmaeus*	1	Anura	Craugastoridae	No data	Positive	Familiar-López (2010) and Basanta et al. (2021a)
*Craugastor rhodopis*	2	Anura	Craugastoridae	No data	Positive	Murrieta-Galindo et al. (2014) and Basanta et al. (2021a)
*Craugastor* sp.	3	Anura	Craugastoridae	No data	Positive	Basanta et al. (2021a)
*Eleutherodactylus* sp.	1	Anura	Eleutherodactylidae	No data	Positive	Basanta et al. (2021a)
*Dryophytes arenicolor*	2	Anura	Hylidae	Tolerant	Positive	Basanta et al. (2021a)
*Hyliola cadaverina*	3	Anura	Hylidae	Tolerant	Positive	Peralta-García et al. (2018) and Basanta et al. (2021a)
*Hyliola regilla*	22	Anura	Hylidae	Tolerant	Positive	Luja et al. (2012), Peralta-García et al. (2018) and Basanta et al. (2021a)
*Plectrohyla matudai*	1	Anura	Hylidae	Tolerant	Positive	Muñoz Alonso (2010) and Basanta et al. (2021a)
*Plectrohyla* sp.	1	Anura	Hylidae	Tolerant	Positive	Basanta et al. (2021a)
*Ptychochyla zophodes*	1	Anura	Hylidae	Tolerant	Positive	Basanta et al. (2021a)
*Ptycohyla* sp.	2	Anura	Hylidae	Tolerant	Positive	Basanta et al. (2021a)
*Scinax staufferi*	5	Anura	Hylidae	Tolerant	Positive	Basanta et al. (2021a)
*Smilisca baudinii*	2	Anura	Hylidae	Tolerant	Positive	Familiar-López (2010), Muñoz Alonso (2010) and Basanta et al. (2021a)
*Tlalocohyla loquax*	1	Anura	Hylidae	Tolerant	Positive	Muñoz Alonso (2010)
*Tripion spinosus*	1	Anura	Hylidae	Tolerant	Positive	Basanta et al. (2021a)
*Leptodactylus melanonotus*	3	Anura	Leptodactylidae	No data	Positive	Cortés-García (2014), Basanta et al. (2021a) and Basanta et al. (2021b)
*Agalychnis callydrias*	3	Anura	Phyllomedusidae	No data	Positive	Basanta et al. (2021a)
*Agalychnis dacnicolor*	4	Anura	Phyllomedusidae	No data	Positive	García-Feria et al. (2017), Basanta et al. (2021a) and Basanta et al. (2021b)
*Agalychnis moreletii*	5	Anura	Phyllomedusidae	No data	Positive	Frías-Alvarez et al. (2008), Basanta et al. (2021a) and Basanta et al. (2021b)
*Rana berlandieri*	3	Anura	Ranidae	Tolerant	Positive	Murrieta-Galindo et al. (2014), Feria, Brousset & Olivares (2019), Hernández-Martínez et al. (2019) and Basanta et al. (2021a)
*Rana catesbeiana*	1	Anura	Ranidae	Tolerant	Positive	Hernández-Martínez et al. (2019) and Basanta et al. (2021a)
*Rana draytonii*	1	Anura	Ranidae	Tolerant	Positive	Peralta-García et al. (2018) and Basanta et al. (2021a)
*Rana montezumae*	12	Anura	Ranidae	Tolerant	Positive	Frías-Alvarez et al. (2008), García-Feria et al. (2017) and Basanta et al. (2021a)
*Rana neovolcanica*	6	Anura	Ranidae	Tolerant	Positive	Frías-Alvarez et al. (2008) and Basanta et al. (2021a)
*Rana sierramadrensis*	2	Anura	Ranidae	Tolerant	Positive	Familiar-López (2010) and Basanta et al. (2021a)
*Rana* sp.	7	Anura	Ranidae	Tolerant	Positive	Basanta et al. (2021a)
*Ambystoma altamirani*	78	Caudata	Ambystomatidae	Tolerant	Positive	Frías-Alvarez et al. (2008) and Basanta et al. (2021b)
*Ambystoma andersoni*	30	Caudata	Ambystomatidae	Tolerant	Positive	Basanta, Rebollar & Parra-Olea (2019)
*Ambystoma dumerilii*	16	Caudata	Ambystomatidae	Tolerant	Positive	Basanta, Rebollar & Parra-Olea (2019)
*Ambystoma flavipiperatum*	1	Caudata	Ambystomatidae	Tolerant	Positive	Basanta, Rebollar & Parra-Olea (2019)
*Ambystoma granulosum*	38	Caudata	Ambystomatidae	Tolerant	Positive	Frías-Alvarez et al. (2008)
*Ambystoma mexicanum*	27	Caudata	Ambystomatidae	Tolerant	Positive	García-Feria et al. (2017)
*Ambystoma ordinarium*	100	Caudata	Ambystomatidae	Tolerant	Positive	Basanta et al. (unpublished data)
*Ambystoma rivulare*	2	Caudata	Ambystomatidae	Tolerant	Positive	Frías-Alvarez et al. (2008) and Basanta, Rebollar & Parra-Olea (2019)
*Ambystoma taylori*	24	Caudata	Ambystomatidae	Tolerant	Positive	Basanta et al. (unpublished data)
*Ambystoma velasci*	60	Caudata	Ambystomatidae	Tolerant	Positive	Frías-Alvarez et al. (2008) and García-Feria et al. (2017)
*Aquiloeurycea cafetalera*	1	Caudata	Plethodontidae	Lethal	Positive	Parra-Olea et al. (unpublished data)
*Bolitoglossa franklini*	1	Caudata	Plethodontidae	Lethal	Positive	Basanta et al. (2021a)
*Bolitoglossa occidentalis*	3	Caudata	Plethodontidae	Lethal	Positive	Basanta et al. (2021a)
*Bolitoglossa platydactyla*	1	Caudata	Plethodontidae	Lethal	Positive	Basanta et al. (2021a)
*Chiropterotriton totonacus*	1	Caudata	Plethodontidae	Lethal	No data	No data
*Dendrotriton xolocalcae*	2	Caudata	Plethodontidae	Lethal	Positive	Muñoz Alonso (2010)
*Parvimolge townsendi*	1	Caudata	Plethodontidae	Lethal	Positive	Parra-Olea et al. (unpublished data)
*Pseudoeurycea leprosa*	2	Caudata	Plethodontidae	Lethal	Positive	Van Rooij et al. (2011), Mendoza-Almeralla et al. (2016) and Basanta et al. (2021a)
*Pseudoeurycea longicauda*	1	Caudata	Plethodontidae	Lethal	Positive	Van Rooij et al. (2011)

**Notes.**

Fifth column indicates the predicted *Bsal* vulnerability based on infection trials performed in species from the same amphibian family (Martel et al., 2014; North American Bsal Task Force, 2022). Last two columns show data on *Bd* infection detected in Mexico.

## Discussion

This is the first study that evaluates the presence of *Bsal* in amphibian species across different regions of Mexico. We did not detect *Bsal* in any of the amphibian skin samples from captive or wild individuals from North, Central and Southern Mexico. The non-detection of *Bsal* in those areas suggests that to date, *Bsal* has not yet arrived to these areas. In agreement with our results, previous studies have not detected *Bsal* in the USA and Northern Mexico (Bales et al., 2015; Klocke et al., 2017; Parrott et al., 2017; Newman et al., 2019; Hardman et al., 2020; Waddle et al., 2020; Hill et al., 2021), which could indicate that *Bsal* is still absent in North America, or that *Bsal* is at very low prevalence within these populations and has a low detection probability. Because the risk of this pathogen is high for amphibians and its arrival to America is imminent, we highlight the importance of continuous surveillance of *Bsal* in areas of potential risk and new areas where the pathogen arrival is more likely to occur.

To date, *Bsal* detections from animals in the wild remain restricted to Europe and Asia (Martel et al., 2014; Laking et al., 2017; Yuan et al., 2018; Lötters et al., 2020), and amphibians from the pet trade from Asia have been found infected by *Bsal* (Cunningham et al., 2015; Nguyen et al., 2017; Yuan et al., 2018; Martel et al., 2020). Thus, individuals from trade markets are considered as the principal potential source for the spread of this pathogen to naïve areas (Gray et al., 2015). In our study, we sampled captive individuals of *Ambystoma mexicanum*. This species is one of the most traded amphibians worldwide (Carpenter et al., 2014), and the potential presence of *Bsal* in this species could threaten not only amphibians from Mexico but also from other countries that have a high amphibian trade such as the USA and Canada. Future surveillance should include the screening of amphibians that were potentially imported.

Considering the high amphibian richness present in Mexico, the high number of species in some category of risk, and the potential vulnerability in plethodontid species from laboratory infection trials, the introduction of *Bsal* into the country should be considered a major threat to amphibian biodiversity. Our survey includes regions that have a high amphibian taxonomic and functional diversity (Ochoa-Ochoa et al., 2020; García-Rodríguez et al., 2022), and/or have a high or moderate *Bsal* habitat suitability: the Sierra Madre Oriental, the extreme eastern Trans-Mexican Volcanic Belt, southeastern Sierra Madre del Sur, mountains of Chiapas in Mexico (García-Rodríguez et al., 2022). One example are the Mexican bolitoglossine salamanders *Chiropterotriton* spp. and *Aquiloeurycea cephalica* which are highly vulnerable to *Bsal* as evidenced by their high mortality in experimental infections (North American *Bsal* Task Force, 2022). Thus, the arrival of *Bsal* could cause decreases in populations and could dramatically reduce salamander bolitoglossine biodiversity.

Moreover, we sampled 41 of the 103 amphibian species that have tested positive to *Bd* infection in Mexico (Table 1; López-Velázquez, 2018; Basanta et al., 2021a). The potential co-infection of *Bd* and *Bsal* to these species could affect them severely (Longo, Fleischer & Lips, 2019). We suggest continuing the surveillance and increasing the efforts in the areas and species with high risk (*i.e.,* areas with high *Bsal* suitability, high amphibian richness, and *Bd* presence), however, it is also important to start strict *Bsal* surveillance and monitoring at all entry points for exotic species for trade.

Understanding the potential entry sites and invasion routes of *Bsal* may help in designing efficient surveillance and conservation strategies. Pathogen transmission could be influenced by the amphibian species-specific *Bsal* susceptibility to infection. Thus, knowledge of species susceptibility to *Bsal* and information on its spatial transmission (Kearney & Porter, 2009; Malagon et al., 2020) may help predicting potential spreading routes in the country if it were to be introduced. Previous experimental studies have shown that salamanders infected by *Bsal* show clinical signs of chytridiomycosis, whereas anurans were asymptomatic and may act as potential reservoirs of the pathogen (Martel et al., 2014; North American *Bsal* Task Force, 2022). In contrast, a recent study found that the Cuban treefrog *Osteopilus septentrionalis* is highly susceptible to *Bsal* disease (Towe et al., 2021), demonstrating that infections of *Bsal* could also affect anuran species under specific scenarios. Experimental research on host *Bsal* susceptibility in Mexican anuran and salamander species is urgently needed.

## Conclusions

The emergence of infectious diseases such as chytridiomycosis in amphibians poses a major threat to wildlife populations (Skerratt et al., 2007; Martel et al., 2013; Fisher & Garner, 2020). To date, there are no effective treatments for *Bsal* infections in natural populations. The non-detection of *Bsal* in our study suggests that to date *Bsal* has not yet arrived in these regions in Mexico. The major challenges are the design of *in situ* disease containment and mitigation post *Bsal* arrival (Canessa et al., 2018; Thomas et al., 2019). Control strategies should focus on preventive measures to reduce the introduction risk such as the implementation of biosecurity measures in amphibian trade, and surveillance in areas of potential introduction and high risk. For example, the United States Fish and Wildlife and the government of Canada restricted salamander imports from 2016 and 2017 respectively, which has likely prevented the arrival of *Bsal* to these countries (https://www.ecfr.gov/current/title-50/part-16; Government of Canada, 2017; North American *Bsal* Task Force, 2022). It is imperative for a swift legislative change to prevent pathogen spread and implement a strategic plan to prevent and control *Bsal* invasion in Mexico (North American *Bsal* Task Force, 2022). For this, it is urgently necessary to establish a working network between Mexican authorities, research groups, pet shops, and local communities to report any signs of disease in any organism to stop the possible spread of *Bsal* in Mexico.

##  Supplemental Information

10.7717/peerj.14117/supp-1 Table S1 Skin swab samples taken for the detection of *Batrachochytrium salamandrivorans* (Bsal)

## Additional Information and Declarations

### Field Study Permissions

The following information was supplied relating to field study approvals (*i.e.*, approving body and any reference numbers): Collection permits were provided by the Secretaría del Medio Ambiente y Recursos Naturales (SEMARNAT): SGPA/DGVS/00947/16, SGPA/DGVS/03038/17, SGPA/DGVS/003513/18, SGPA/DGVS/002176/18, SPA-ENS/305/18, SEDUMA/SP/2738/2018, RBMM. DIREC/208/18, UNAM JJBIB/54/2017, SGPA/DGVS/5673/19, and SGPA/DGVS/02770/21.

### Data Availability

The following information was supplied regarding data availability: The raw data is available as Supplemental File.
